# Association of single nucleotide polymorphisms in *MTHFR* and *ABCG2* with the different efficacy of first-line chemotherapy in metastatic colorectal cancer

**DOI:** 10.1007/s12032-013-0802-6

**Published:** 2013-12-13

**Authors:** Jing Zhao, Wenhua Li, Dan Zhu, Qihe Yu, Zhe Zhang, Menghong Sun, Sanjun Cai, Wen Zhang

**Affiliations:** 1 Department of Medical Oncology, Fudan University Shanghai Cancer Center, Shanghai, China; 2 Department of Pathology, Fudan University Shanghai Cancer Center, Shanghai, China; 3 Department of Colorectal Surgery, Fudan University Shanghai Cancer Center, Shanghai, China; 4 Department of Oncology, Shanghai Medical College, Fudan University, Shanghai, 200032 China

**Keywords:** Colorectal neoplasms, Oxaliplatin, Irinotecan, Polymorphisms, *MTHFR*, *ABCG2*

## Abstract

Either oxaliplatin- or irinotecan-containing regimen could receive a good effectiveness in patients with metastatic colorectal cancer as the first-line chemotherapy, but not all patients would benefit from the treatment they have received. This study was to investigate the role of single nucleotide polymorphisms (SNPs) of methylenetetrahydrofolate reductase (*MTHFR*) and ATP-binding cassette sub-family G member 2 (*ABCG2*) in selecting the most appropriate treatment for individual patients. Ninety-two metastatic colorectal cancer patients treated with first-line 5-fluoropyrimidine (5-FU), leucovorin, and oxaliplatin (FOLFOX), capecitabine, and oxaliplatin (XELOX) and sixty-two patients receiving 5-FU, leucovorin, and irinotecan (FOLFIRI) were reviewed. The SNPs of *MTHFR* and *ABCG2* were detected using gene sequencing method after DNA PCR amplification, which was extracted from peripheral blood karyocytes. Clinical characteristics and gene polymorphisms were evaluated in univariate and multivariate analysis as predictive factors for response rate (RR) and progression-free survival (PFS). In patients bearing 2–4 genotypes of *MTHFR* 677C/C, *MTHFR* 1298 A/C or C/C, *ABCG2* 34G/G, and *ABCG2* 421C/A or A/A, those who received oxaliplatin-based chemotherapy achieved a higher RR (41.7 vs. 18.8 %, *P* = 0.027) and longer median PFS (mPFS) than irinotecan-based therapy [8.9 vs. 7.1 m, FOLFIRI: hazard ratio (HR) = 1.722, 95 % confidence interval (CI) 1.026–2.892, *P* = 0.040, compared with FOLFOX/XELOX]; on the contrary, patients carrying 0 or 1 above genotype exhibited better outcomes after receiving FOLFIRI chemotherapy (mPFS: 9.3 vs. 6.4 m, FOLFIRI: HR = 0.422, 95 % CI 0.205–0.870, *P* = 0.019, compared with FOLFOX/XELOX). Combination of SNPs with *MTHFR* and *ABCG2* may play a role in helping clinicians to select first-line chemotherapy for patients with metastatic colorectal cancer.

## Introduction

Colorectal cancer (CRC) is one of the commonly diagnosed cancers among patients, which ranks the third for males and the second for females worldwide. For the fatality rate, it ranks the fourth and the third for males and females, respectively [1].

Systemic combination chemotherapy is the mainstay of the treatment for patients with metastatic colorectal cancer (mCRC). Treatment for patients with mCRC has evolved significantly over the last 10 years with the use of new active cytotoxic agents, including oxaliplatin and irinotecan plus targeted monoclonal antibodies bevacizumab, cetuximab, and panitumumab; 5-fluoropyrimidine (5-FU), leucovorin (CF), and oxaliplatin (FOLFOX), capecitabine, and oxaliplatin (XELOX) or 5-FU, CF, and irinotecan (FOLFIRI) are the standard chemotherapy treatment for metastatic colorectal cancer in practice with equivalent treatment effectiveness [2–4]. However, some patients do not necessarily benefit from the treatment they have received, but are exposed to the adverse effects nonetheless [5]. Therefore, it is of great importance to identify biomarkers that could help select the optimum regimen for each patient.

Genetic polymorphisms in drug target genes, genes encoding DNA repair enzymes, and detoxification pathways may influence the activity of the drug. Single nucleotide polymorphism (SNP) refers to the DNA sequence polymorphisms caused by single nucleotide variations occurred at the genomic level with the probability greater than 1 %, which sometimes can affect the expression or activity of its encoded protein and therefore affects its function. With respect to the cancer treatment, SNP would be related to different therapeutic efficacy and adverse reactions.

As the common drug in FOLFOX, XELOX, and FOLFIRI regimens, 5-FU or its derivate remains the basis for colorectal cancer chemotherapy. Optimal cytotoxicity of fluoropyrimidines requires elevated 5, 10-methylenetetrahydrofolate (CH2FH4) tumoral concentrations, controlled by the methylenetetrahydrofolate reductase (MTHFR) enzyme, which irreversibly converts CH2FH4 into 5-methyltetrahydrofolate. The *MTHFR* gene is subject to several polymorphisms, of which the 677C>T and 1298A>C SNPs are the two most commonly linked with altered enzyme activity. Accordingly, experimental data have shown that rare *MTHFR* variants in position 677 and 1298 are more sensitive to 5-FU [6–9]. However, results of clinical data do not concord regarding the influence of *MTHFR* genotype on 5-FU responsiveness or patients’ survival [10–12].

Breast cancer resistance protein (BCRP/ABCG2) is an ATP-binding cassette transporter, which may directly cause resistance of cancer cells by active efflux of anticancer drugs. It has been found that there are more than 40 SNPs in *ABCG2* gene, the most common two of which are the 421C>A in exon 5 and 34G>A in exon 2. Numerous in vitro studies have shown that 421A cells have a reduced resistance to irinotecan and its active product SN-38 [13, 14], while the polymorphisms of 421C>A in *ABCG2* gene has no effect on the pharmacokinetics of hydrochloric acid irinotecan based on the blood sample from 84 European patients [15]. As for *ABCG2* 34G>A, Mcleod [16] reported at the 2008 ASCO annual meeting that this polymorphism was associated with relative susceptibility to FOLFOX and resistance to FOLFIRI (*P* < 0.013, Caucasians only). The research results remind us that the respective benefit populations of different chemotherapy regimens could be distinguished through the study of single nucleotide polymorphisms. If this conclusion could be confirmed by more researches, this SNP may provide evidence for selecting FOLFOX or FOLFIRI therapy for patients with advanced colorectal patients.

In the present study, we detected the SNPs in *MTHFR* 677C>T, 1298A>C, *ABCG2* 34G>A, and 421C>A in patients with mCRC who had received FOLFOX/XELOX or FOLFIRI as first-line chemotherapy, and combined with the clinical features, we investigated the potential markers for selecting the first-line chemotherapy.

## Methods

### Patients

We retrospectively collected the data for advanced colorectal cancer patients who received first-line standard FOLFOX, XELOX, or FOLFIRI regimen between January 2009 and May 2011 at Fudan University Shanghai Cancer Center. Subjects eligible for this study must meet the following criteria: (1) pathologically confirmed colorectal adenocarcinoma with unrespectable and appraisable metastasis or relapse; (2) must have received first-line FOLFOX, XELOX, or FOLFIRI chemotherapy for at least 2 cycles; (3) age between 18 and 75; (4) ECOG 0-1; and (5) peripheral blood sample was reserved in our tissue bank. Written informed consent was obtained from all patients as well.

### Genotyping

Genomic DNA was isolated from whole blood using the FUJIFILM DNA extraction kit. Based on previous published studies, the single nucleotide polymorphisms selected for testing were *MTHFR* 677C>T (rs1801133, Ala 222 Val) and 1298A>C (rs1801131, Glu 428 Ala), and *ABCG2* 34G>A (rs2231137, Val 12 Met) and 421C>A (rs2231142, Gln 141 Lys). Genotyping for the SNPs was determined using pyrosequencing. Primers used were F, 5′-GGAAGGTGCAAGATCAGAGC-3′ and R, 5′-CTGGGAAGAACTCAGCGAAC-3′ for amplification of codon 222 of *MTHFR*; F, 5′-CCAGACCAAAGAGTTACATCTACCG-3 and R, 5′-CTTACCCTTCTCCCTTTGCCA-3′ for codon 428 of *MTHFR*; F, 5′-TTCCAAGTTGTGCCTGTC-3′ and R, 5′-AAGCCATTGGTGTTTCC-3′ for codon 12 of *ABCG2*; and F, 5′-GGATGATGTTGTGATGGGCACTCT-3′ and R, 5′-GGAAAGCAACCATTTTTGACCATAC-3′ for codon 141 of *ABCG2*.

### Statistics

According to RECIST (version 1.1), the response to treatment was assessed by clinical and radiologic examination using CT scan or MRI of the chest, abdomen, and pelvis. Objective response rate (RR) refers to the percentage of patients having complete response (CR) or partial response (PR). Patients with stable disease (SD) or progressive disease (PD) are defined as nonresponders. Progression-free survival (PFS) is defined as the time interval from the start of first-line chemotherapy to first disease progression or death from any cause if disease progression does not occur. Alive patients without progression will be censored at the last follow-up.

PFS was estimated by the Kaplan–Meier method. The relationship between prognostic factors and PFS was explored using log-rank test and Cox regression model for univariate and multivariate analyses, respectively. Pearson’s *χ* ^2^ test or Fisher’s exact test was used to assess the association between predictive factors and RR, while for multivariate analyses, binary logistic regression model was employed. *P* values were two-tailed for all the tests, and statistical significance was set as *P* < 0.05. Analyses were conducted using SPSS 17.0.

## Results

### Patient characteristics

Of all 154 patients enrolled, ninety (58 %) patients were male, and 64 (42 %) were female, with a median age of 56 (range 30–75). All treated patients had an ECOG PS of 0 or 1. Ninety-two patients were diagnosed with rectal cancer and 62 with colon cancer. Radical resection of the primary tumor had been performed in 86 % of the patients (*n* = 132). Synchronous metastasis occurred in 78 patients, and metachronous metastasis or relapse occurred in 76 patients. Ninety-five patients had a single metastatic organ, and 59 had more than one metastatic organs. One patient achieved CR (1 %), 48 patients achieved PR (31 %), 75 patients had SD (49 %), and 29 had PD (19 %), with a RR of 32 %. Until March 12, 2012, one hundred and thirty-seven (89 %) patients have progressed, with the mPFS of 8.1 months [95 % confidence interval (CI) 6.9–9.3]. Among them, 92 patients received oxaliplatin-containing regimen (FOLFOX or XELOX), with a RR of 35.9 % and mPFS of 8.3 months; the rest 62 patients were treated with FOLFIRI chemotherapy, with a RR of 26.2 % and mPFS of 8.1 months. No significant differences were observed for either RR (*P* = 0.211) or PFS (*P* = 0.443) between the two regimens. Table 1 presents the demographic characteristics of the participants enrolled in the study. There was no significant difference in age, gender, primary site of tumor, radical resection of primary tumor, and the number of metastatic organs, between two groups of patients treated with different regimens, except the time of metastasis. Of all 154 patients, each of the genotypes of *MTHFR* 1298A>C and ABCG2 34G>A was not available for one patient, respectively. Table 2 illustrates the distributions of genotypes for the SNPs.

**Table 1:** The characteristics of FOLFOX/XELOX and FOLFIRI groups

Characteristic	FOLFOX/XELOX *n* (%)	FOLFIRI *n* (%)	*P* value
Age (year)			
Mean ± SD	56.07 ± 9.6	54.98 ± 9.8	0.826
Gender			
Male	56 (60.9)	34 (54.8)	0.456
Female	36 (39.1)	28 (45.2)	
Primary site			
Rectum	54 (58.7)	38 (61.3)	0.747
Colon	38 (41.3)	24 (38.7)	
Radical resection of primary site			
Yes	78 (84.8)	54 (87.1)	0.687
No	14 (15.2)	8 (12.9)	
Number of metastatic organs			
Single	59 (64.1)	36 (58.1)	0.448
Multiple	33 (35.9)	26 (41.9)	
Time of metastasis			
Heterochrony	29 (31.5)	47 (75.8)	**0.000**
Synchrony	63 (68.5)	15 (24.2)	

Bold value indicates statistical significance

*FOLFOX* 5-fluoropyrimidine, leucovorin, and oxaliplatin, *XELOX* capecitabine and oxaliplatin, *FOLFIRI* 5-fluoropyrimidine, leucovorin, and irinotecan, *SD* standard deviation

**Table 2:** The genotypes distributions of *MTHFR* and *ABCG2* genes

Genotype	Case/n	Percentage (%)
*MTHFR* 677		
C/C	55	35.7
C/T	77	50.0
T/T	22	14.3
*MTHFR* 1298		
A/A	101	66.0
A/C	44	28.8
C/C	8	5.2
*ABCG2* 34		
G/G	71	46.4
G/A	58	37.9
A/A	24	15.7
*ABCG2* 421		
C/C	60	39.0
C/A	78	50.6
A/A	16	10.4

*MTHFR* methylenetetrahydrofolate reductase, *ABCG2* ATP-binding cassette sub-family G member 2

### Predictive factors

**Fig. 1:**
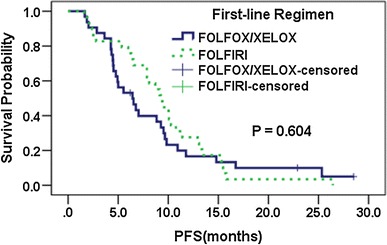
Kaplan–Meier estimation of PFS by the *first-line* chemotherapy in low-score group

**Fig. 2:**
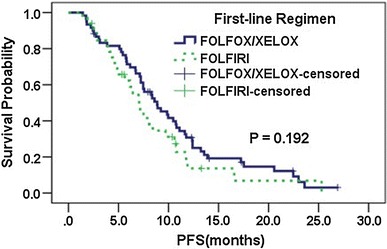
Kaplan–Meier estimation of PFS by the *first-line* chemotherapy in high-score group

For patients receiving FOLFOX/XELOX chemotherapy, RR was higher and PFS was longer in patients carrying *MTHFR* 677C/C, 1298 A/C or C/C, *ABCG2* 34G/G, or 421C/A or A/A; in contrast, for patients treated with FOLFIRI, RR was lower and PFS was shorter in those genotypes, except that there was no difference in PFS between patients bearing *MTHFR* 677C/C and a genotype containing T. However, there were almost no significant differences, except *ABCG2* 421C>A in RR for patients treated with FOLFOX/XELOX (seen in Table 3). Based on these results, we defined *MTHFR* 677C/C, *MTHFR* 1298 A/C or C/C, *ABCG2* 34G/G, and *ABCG2* 421 C/A or A/A as the favorable genotypes for FOLFOX/XELOX regimen, and each was defined as 1 point each, while the opposite genotype was 0 point each. A score of 0–4 was calculated for each patient. Two groups were identified according to the score: a low-score group (0–1 point), and high-score group (2–4 points). The univariate analysis showed that in the low-score group, patients treated with FOLFIRI had a longer mPFS than those administrated by FOLFOX/XELOX (9.3 vs. 6.4 months, *P* = 0.604, Fig. 1), and PFS was significantly associated with radical resection of primary lesion, the number of metastatic organs, and the time of metastasis, while in the high-score group, patients receiving FOLFIRI had a shorter mPFS than those treated with FOLFOX/XELOX (7.1 vs. 8.9 months, *P* = 0.192, Fig. 2), and PFS was significantly associated with radical resection of primary lesion. These three variables were introduced in the multivariate analysis, along with the first-line chemotherapy. After being adjusted for radical resection of primary lesion, the number of metastatic organs, and the time of metastasis, patients treated with FOLFIRI were associated with a 57.8 % reduced risk of disease progression [adjusted hazard ratio (HR) = 0.422, 95 % CI 0.205–0.870, *P* = 0.019] compared with FOLFOX/XELOX, while in the high-score group, patients receiving FOLFIRI were associated with a 72.2 % increased risk of disease progression (adjusted HR = 1.722, 95 % CI 1.026–2.892, *P* = 0.040) (seen in Tables 4 and 5). As for RR, the univariate analysis showed in the low-score group, patients treated with FOLFIRI had a higher RR than those treated with FOLFOX/XELOX (55.8 vs. 44.4, *P* = 0.417), while in the high-score group, the RR was significantly higher in patients treated with FOLFOX/XELOX (41.7 vs. 18.8, *P* = 0.027). However, the multivariate analysis showed no significant differences.

**Table 3:** The relationship between single SNP and PFS in different chemotherapy regimens

Genotype	RR (%)	FOLFOX/XELOX group			FOLFIRI group			*P* value
		*P* value	mPFS (m)	*P* value	RR (%)	*P* value	mPFS (m)	
*MTHFR*-677								
C/C	43.6	0.185	10.0	0.434	18.8	0.428	8.1	0.178
C/T + T/T	30.2		7.0		28.3		8.1	
*MTHFR*-1298								
A/A	34.5	0.857	7.6	0.763	30.2	0.231	8.7	0.199
A/C + C/C	36.4	0.108	8.4	0.472	15.8	0.357	7.2	0.220
*ABCG2*-34								
G/G	44.4		8.9		19.2		6.3	
G/A + A/A	28.3		6.7		30.6		9.3	
*ABCG2*-421								
C/C	21.6	0.019	6.6	0.212	30.4	0.561	9.1	0.757
C/A + A/A	45.5		8.9		23.1		7.5	

*SNP* single nucleotide polymorphism, *PFS* progression-free survival, *FOLFOX* 5-fluoropyrimidine, leucovorin, and oxaliplatin, *XELOX* capecitabine and oxaliplatin, *FOLFIRI* 5-fluoropyrimidine, leucovorin, and irinotecan, *MTHFR* methylenetetrahydrofolate reductase, *ABCG2* ATP-binding cassette sub-family G member 2

**Table 4:** The multivariate analysis of PFS in low-score group

Variable	HR	95 % CI	*P* value
First-line FOLFIRI	0.422	0.205–0.870	**0.019**
Synchronous metastasis	0.238	0.094–0.600	**0.002**
Radical resection of primary site	0.171	0.061–0.480	**0.001**
Multiple metastatic organs	0.823	0.435–1.554	0.548

Bold values indicate statistical significance

*PFS* progression-free survival, *HR* hazard ratio, *CI* confidence interval, *FOLFIRI* 5-fluoropyrimidine, leucovorin, and irinotecan

**Table 5:** The multivariate analysis of PFS in high-score group

Variable	HR	95 % CI	*P* value
First-line FOLFIRI	1.722	1.026–2.892	**0.040**
Synchronous metastasis	1.757	1.028–3.003	**0.039**
Radical resection of primary site	0.726	0.345–1.527	0.399
Multiple metastatic organs	1.424	0.836–2.426	0.194

Bold values indicate statistical significance

*PFS* progression-free survival, *HR* hazard ratio, *CI* confidence interval, *FOLFIRI* 5-fluoropyrimidine, leucovorin, and irinotecan

## Discussion

While the fatality rate for colorectal cancer has been decreasing in several developed countries, the rate continues to increase in many developing countries with limited resources and health infrastructure [1]. Even with the application of the new drugs of oxaliplatin and irinotecan, still nearly half of the advanced colorectal patients could not benefit from the chemotherapy they received. Even though FOLFOX, XELOX, and FOLFIRI have similar efficacy on either RR, PFS, or OS, until recently, little is known about how to select the first-line chemotherapy for patients with metastatic colorectal cancer.

We identified two groups suitable for different regimens depending on the SNPs of *MTHFR* and *ABCG2.* Patients carrying 0–1 of *MTHFR* 677C/C, *MTHFR* 1298A/C or C/C, *ABCG2* 34G/G, and *ABCG2* 421C/A or A/A genotypes had higher RR and longer PFS when treated with FOLFIRI regimen as the first-line chemotherapy, while patients carrying 2–4 of above genotypes had higher RR and longer PFS after treated with FOLFOX or XELOX. To our knowledge, this is the first study to explore the predictive biomarkers to select the first-line regimen for patients with mCRC published to date.

In this study, we analyzed the data for 154 patients with advanced colorectal cancer who had received FOLFOX, XELOX, or FOLFIRI as the first-line chemotherapy. In the FOLFOX/XELOX group, the RR was 36 %, and the PFS was 8.3 months, while in the FOLFIRI group, the RR and PFS were 26.2 % and 8.1 months, respectively. No significant differences were observed between the two groups, which was similar with the results reported previously. There have been no predictive biomarkers for choosing the first-line chemotherapy for patients. In our study, we investigated the predictive role of SNPs of *MTHFR* and *ABCG2* for selecting the first-line treatment. MTHFR irreversibly converts CH2FH4 into CH3FH4, and intracellular CH2FH4 concentration is mainly controlled by *MTHFR* [17]. In agreement, experimental [18] and clinical [19] studies have established that optimal 5-FU cytotoxicity requires elevated CH2FH4 tumoral concentrations. A study by Cohen et al. [7] is the first that reported a link between the *MTHFR* genotype and tumor response to 5-FU-based chemotherapy, among which of 43 metastatic colorectal cancer patients receiving exclusive 5-FU therapy, all five 677TT patients responded to the treatment, whereas the response rate was approximately 50 % in 677CC patients. Terrazzino et al. [8] found that in rectal cancer patients receiving 5-FU-based chemotherapy and radiotherapy, patients not carrying the *MTHFR* 677T-1298A haplotype exhibited a higher response rate than patients with the *MTHFR* 677T-1298A haplotype (*P* = 0.002). By contrast, most studies containing advanced colorectal cancer patients receiving 5-FU associated with irinotecan or oxaliplatin failed to show a link between the two *MTHFR* polymorphisms (677C>T and 1298A>C) and RR or survival [11, 20, 21]. Thus, some researchers considered that the predictive role of *MTHFR* polymorphisms on 5-FU responsiveness has been reported in studies where 5-FU was the central drug; however, it would appear that the presence of oxaliplatin or irinotecan in current 5-FU-based treatment could blur the influence of *MTHFR* polymorphisms on the treatment outcomes [22]. Exact mechanism remains unclear, which is needed elucidating by further studies.

ABCG2 is a member of the ABC transporter family, which was first cloned from doxorubicin-resistant human MCF-7 breast cancer cells and was named breast cancer resistance protein (BCRP) [23]. ABCG2 is a transmembrane transporter that carries out many chemotherapy drugs out of cells, including CPT-11 and its active metabolite SN-38 [24]. The SNPs of *ABCG2* affect the pharmacokinetics of many drugs by affecting either expression of ABCG2, the activity of ABCG2, or the transport efficiency of substrate. The data presented by Kobayashi suggested that the 421C>A variant in the *BCRP* gene, a common SNP in both Japanese and Caucasian populations, alters protein levels and lowers the activity of its ATP enzyme, thus increasing the sensitivity of anticancer drugs. Vitro studies indicated that 421A-cells have a lower drug resistance to CPT-11 and SN-38 [13, 14]. The report presented at 2008 ASCO annual meeting, which indicated that ABCG2 34G>A was associated with relative susceptibility to FOLFOX and resistance to FOLFIRI [16], aroused us to analyze the predictive value of SNP for different chemotherapy regimens.

Our study found that single SNP has no predictive role on efficacy or survival for the patients treated with FOLFOX/XELOX or FOLFIRI. However, when several SNPs combined, favorable patients for different regimens can be separated: Patients carrying 2–4 of MTHFR 677C/C, MTHFR 1298 A/C or C/C, ABCG2 34G/G, and ABCG2 421C/C or A/A may benefit more from FOLFOX/XELOX regimen as the first-line chemotherapy. On the other side, patients carrying 0–1 above genotype may get more benefits from the chemotherapy of FOLFIRI.

Conclusively, the results of the study indicated that the combination of SNPs may play a role in determining the first-line chemotherapy for patients. However, the limitations of the retrospective study, mainly small sample size, could affect final results. Therefore, these results need to be validated in a larger prospective study further to seek the predictive scale to determine the best treatment for patients, combined with other biomarkers, such as microRNA, mRNA, or protein. To elucidate the mechanism underlying the polymorphisms and chemotherapy efficacy, further functional evaluations are needed.

## References

[1] Jemal A, Bray F, Center MM, Ferlay J, Ward E, Forman D (2011). Global cancer statistics. CA Cancer J Clin.

[2] Tournigand C, Andre T, Achille E, Lledo G, Flesh M, Mery-Mignard D, Quinaux E, Couteau C, Buyse M, Ganem G, Landi B, Colin P, Louvet C, de Gramont A (2004). FOLFIRI followed by FOLFOX6 or the reverse sequence in advanced colorectal cancer: a randomized GERCOR study. J Clin Oncol.

[3] Colucci G, Gebbia V, Paoletti G, Giuliani F, Caruso M, Gebbia N, Carteni G, Agostara B, Pezzella G, Manzione L, Borsellino N, Misino A, Romito S, Durini E, Cordio S, Di Seri M, Lopez M, Maiello E, Montemurro S, Cramarossa A, Lorusso V, Di Bisceglie M, Chiarenza M, Valerio MR, Guida T, Leonardi V, Pisconti S, Rosati G, Carrozza F, Nettis G, Valdesi M, Filippelli G, Fortunato S, Mancarella S, Brunetti C (2005). Phase III randomized trial of FOLFIRI versus FOLFOX4 in the treatment of advanced colorectal cancer: a multicenter study of the Gruppo Oncologico Dell’Italia Meridionale. J Clin Oncol.

[4] Ducreux M, Bennouna J, Hebbar M, Ychou M, Lledo G, Conroy T, Adenis A, Faroux R, Rebischung C, Bergougnoux L, Kockler L, Douillard JY (2011). Capecitabine plus oxaliplatin (XELOX) versus 5-fluorouracil/leucovorin plus oxaliplatin (FOLFOX-6) as first-line treatment for metastatic colorectal cancer. Int J Cancer.

[5] Chua W, Kho PS, Moore MM, Charles KA, Clarke SJ (2011). Clinical, laboratory and molecular factors predicting chemotherapy efficacy and toxicity in colorectal cancer. Crit Rev Oncol Hematol.

[6] Jakobsen A, Nielsen JN, Gyldenkerne N, Lindeberg J (2005). Thymidylate synthase and methylenetetrahydrofolate reductase gene polymorphism in normal tissue as predictors of fluorouracil sensitivity. J Clin Oncol.

[7] Cohen V, Panet-Raymond V, Sabbaghian N, Morin I, Batist G, Rozen R (2003). Methylenetetrahydrofolate reductase polymorphism in advanced colorectal cancer: a novel genomic predictor of clinical response to fluoropyrimidine-based chemotherapy. Clin Cancer Res.

[8] Terrazzino S, Agostini M, Pucciarelli S, Pasetto LM, Friso ML, Ambrosi A, Lisi V, Leon A, Lise M, Nitti D (2006). A haplotype of the methylenetetrahydrofolate reductase gene predicts poor tumor response in rectal cancer patients receiving preoperative chemoradiation. Pharmacogenet Genomics.

[9] Boige V, Mendiboure J, Pignon JP, Loriot MA, Castaing M, Barrois M, Malka D, Tregouet DA, Bouche O, Le Corre D, Miran I, Mulot C, Ducreux M, Beaune P, Laurent-Puig P (2010). Pharmacogenetic assessment of toxicity and outcome in patients with metastatic colorectal cancer treated with LV5FU2, FOLFOX, and FOLFIRI: FFCD 2000-05. J Clin Oncol.

[10] Chua W, Goldstein D, Lee CK, Dhillon H, Michael M, Mitchell P, Clarke SJ, Iacopetta B (2009). Molecular markers of response and toxicity to FOLFOX chemotherapy in metastatic colorectal cancer. Br J Cancer.

[11] Ruzzo A, Graziano F, Loupakis F, Rulli E, Canestrari E, Santini D, Catalano V, Ficarelli R, Maltese P, Bisonni R, Masi G, Schiavon G, Giordani P, Giustini L, Falcone A, Tonini G, Silva R, Mattioli R, Floriani I, Magnani M (2007). Pharmacogenetic profiling in patients with advanced colorectal cancer treated with first-line FOLFOX-4 chemotherapy. J Clin Oncol.

[12] Sharma R, Hoskins JM, Rivory LP, Zucknick M, London R, Liddle C, Clarke SJ (2008). Thymidylate synthase and methylenetetrahydrofolate reductase gene polymorphisms and toxicity to capecitabine in advanced colorectal cancer patients. Clin Cancer Res.

[13] Imai Y, Nakane M, Kage K, Tsukahara S, Ishikawa E, Tsuruo T, Miki Y, Sugimoto Y (2002). C421A polymorphism in the human breast cancer resistance protein gene is associated with low expression of Q141 K protein and low-level drug resistance. Mol Cancer Ther.

[14] Morisaki K, Robey RW, Ozvegy-Laczka C, Honjo Y, Polgar O, Steadman K, Sarkadi B, Bates SE (2005). Single nucleotide polymorphisms modify the transporter activity of ABCG2. Cancer Chemother Pharmacol.

[15] de Jong FA, Marsh S, Mathijssen RH, King C, Verweij J, Sparreboom A, McLeod HL (2004). ABCG2 pharmacogenetics: ethnic differences in allele frequency and assessment of influence on irinotecan disposition. Clin Cancer Res.

[16] McLeod HL, Kroetz D, Das S, Giacomini K, Venook AP (2008). Cellular transporter pharmacogenetics in metastatic colorectal cancer: initial analysis of C80203. J Clin Oncol.

[17] Scott J, Weir D (1994). Folate/vitamin B12 inter-relationships. Essays Biochem.

[18] Cheradame S, Etienne MC, Chazal M, Guillot T, Fischel JL, Formento P, Milano G (1997). Relevance of tumoral folylpolyglutamate synthetase and reduced folates for optimal 5-fluorouracil efficacy: experimental data. Eur J Cancer.

[19] Cheradame S, Etienne MC, Formento P, Schneider M, Dassonville O, Demard F, Milano G (1997). Tumoral-reduced folates and clinical resistance to fluorouracil-based treatment in head and neck cancer patients. J Clin Oncol.

[20] Marcuello E, Altes A, Menoyo A, Rio ED, Baiget M (2006). Methylenetetrahydrofolate reductase gene polymorphisms: genomic predictors of clinical response to fluoropyrimidine-based chemotherapy?. Cancer Chemother Pharmacol.

[21] Ruzzo A, Graziano F, Loupakis F, Santini D, Catalano V, Bisonni R, Ficarelli R, Fontana A, Andreoni F, Falcone A, Canestrari E, Tonini G, Mari D, Lippe P, Pizzagalli F, Schiavon G, Alessandroni P, Giustini L, Maltese P, Testa E, Menichetti ET, Magnani M (2008). Pharmacogenetic profiling in patients with advanced colorectal cancer treated with first-line FOLFIRI chemotherapy. Pharmacogenomics J.

[22] Etienne-Grimaldi MC, Francoual M, Formento JL, Milano G (2007). Methylenetetrahydrofolate reductase (MTHFR) variants and fluorouracil-based treatments in colorectal cancer. Pharmacogenomics.

[23] Doyle LA, Yang W, Abruzzo LV, Krogmann T, Gao Y, Rishi AK, Ross DD (1998). A multidrug resistance transporter from human MCF-7 breast cancer cells. Proc Natl Acad Sci USA.

[24] Polgar O, Robey RW, Bates SE (2008). ABCG2: structure, function and role in drug response. Expert Opin Drug Metab Toxicol.

